# Effectiveness of a lymphedema prevention program for patients with breast cancer: A randomized controlled trial based on the Protection Motivation Theory and Information-Motivation-Behavioral Skills Model

**DOI:** 10.1016/j.apjon.2025.100667

**Published:** 2025-02-09

**Authors:** Yuan Wang, Ling Tong, Shan Wang, Weifeng Shi, Dewu Xu

**Affiliations:** a Human Reproductive and Genetic Center, Affiliated Hospital of Jiangnan University, Wuxi, China; b Zhejiang University School of Medicine Sir Run Run Shaw Hospital, Hangzhou, China; c Department of Breast Surgery, Affiliated Hospital of Jiangnan University, Wuxi, China; d Department of Medical Education, Affiliated Hospital of Jiangnan University, Wuxi, China

**Keywords:** Breast cancer, Lymphedema, Nursing interventions, PMT-IMB theory

## Abstract

**Objective:**

This study aims to evaluate the effectiveness of a lymphedema prevention program based on the Protection Motivation Theory and Information-Motivation-Behavioral Skills Model (PMT-IMB) in patients with breast cancer.

**Methods:**

A randomized controlled trial was conducted at a tertiary hospital, enrolling 95 patients treated between December 2022 and June 2023. Participants were randomly assigned to either the control group (*n* ​= ​47), receiving standard nursing care, or the intervention group (*n* ​= ​48), receiving a PMT-IMB-based lymphedema prevention program. The intervention was delivered in hospital settings, with follow-up via WeChat and phone after discharge. Outcomes were assessed using the Lymphedema Risk-Reduction Behavior Checklist (LRRB), Disabilities of the Arm, Shoulder, and Hand (DASH) questionnaire, upper limb circumference measurements, and self-lymphatic drainage records at baseline, 1 month, 3 months, and 6 months post-intervention.

**Results:**

The intervention group demonstrated significantly higher lymphedema prevention behavior scores than the control group after the intervention. Upper limb dysfunction scores improved significantly in the intervention group compared to the control group at three and six months. In the control group, upper limb circumference gradually increased over time, with a significant difference observed at six months. Compliance with self-lymphatic drainage was also significantly better in the intervention group.

**Conclusions:**

The PMT-IMB-based nursing intervention effectively enhances preventive behaviors, reduces lymphedema incidence, improves upper limb function, and increases patient adherence. These findings provide valuable insights for optimizing nursing strategies in lymphedema prevention.

**Trial registration:**

http://www.chictr.org.cn, ChiCTR2300070705.

## Introduction

Epidemiologic surveys report that there will be 19.3 million new cancer cases worldwide in 2020, of which female breast cancer will account for 11.7%, ranking women first in terms of cancer incidence.^1^ Currently, breast cancer is mainly treated through surgery, radiation and chemotherapy.^2^ Patients are susceptible to complications such as bleeding, infection, subcutaneous edema, flap necrosis, and limb dysfunction, with breast cancer-related lymphedema (BCRL) being one of the most common complications after treatment.^3^ Common risk factors for the development of lymphedema include the type of lymph node surgery (axillary dissection versus sentinel lymph node biopsy), the type of treatment (mastectomy versus lumpectomy, radiation therapy, chemotherapy), and the number of positive lymph nodes involved.^4^^,^^5^ Patient characteristics associated with lymphedema include a high body mass index (BMI) and the presence of infection.^6^^,^^7^ BCRL can lead to physical and psychological problems such as limb dysfunction, hypotonia, appearance-related problems, anxiety and depression in patients with breast cancer, thus seriously affecting postoperative recovery and quality of life.^8^ Since breast cancer-associated lymphedema is difficult to cure once it occurs, increasing patient awareness of the risk of lymphedema and focusing on lymphedema prevention interventions is and its necessary to improve the quality of life and survival of patients with breast cancer.^9^

Preventive measures, therapeutic interventions, and continuing care strategies for lymphedema have achieved a relatively advanced status internationally.^10^ A study found that prevention of lymphedema through the use of bioelectrical impedance with tape measurements was effective in preventing lymphedema in patients with breast cancer, but the time-consuming and costly nature of the method has limited its clinical application.^11^ Another study prevented the occurrence of lymphedema and improved patient fatigue and quality of life through aerobic and resistance exercise; however, patient compliance was low and was not effective in preventing lymphedema.^12^ In addition, research evidence emphasizes the important role that early health education and increased motivation for prevention play in preventing the development of lymphedema in patients with breast cancer.^13^ Notably, the survey revealed considerable lack of knowledge and motivation among patients with breast cancer regarding lymphedema prevention, demonstrating the detrimental impact of inadequate professional supervision on postoperative lymphedema prevention.^14^ This suggests the need to strengthen the ability of health care professionals to provide effective, scientific and systematic methods and knowledge of lymphedema prevention to patients with breast cancer, to encourage patients to grasp the importance of lymphedema-related knowledge, to increase their motivation for prevention, and to facilitate the implementation of preventive care processes for patients with breast cancer, thereby reducing the risk of BCRL in patients through early prevention.^15^

The Information-Motivation-Behavioral Skills Model (IMB), first proposed by Fisher scholars in 1992, was initially used for prevention interventions for high-risk behaviors in HIV and is a widely used theoretical model of behavior change by researchers.^16^^,^^17^ The model has three core determinants: information, motivation, and behavioral skills. Protection Motivation Theory (PMT), proposed by Rogers scholars, explores health behaviors from a patient's motivational perspective, with protective behaviors relying on a comprehensive assessment of threat and response and in-depth analysis by the patient of many key factors in health behavior and social cognition, and is an important health psychology and behavior change theory.^18^^,^^19^ Protective motivation theory emphasizes whether individuals engage in protective behaviors and develop protective motives in response to potential threatening factors.

The PMT and IMB models are widely used in the explanation, prediction, and intervention of health behaviors, providing a research foundation for their application in the prevention of lymphedema behaviors. The PMT and IMB models share the goal of promoting health behavior change, and motivation is a key factor in both.^20^ The combination of PMT-IMB theory focuses on an individual's intrinsic motivation and ability to self-accomplish behaviors, aiming to bring the subjective initiative of health behaviors into full play by stimulating an individual's motivation to implement health behaviors.^21^ A study showing the impact of a nursing intervention based on the PMT-IMB theoretical model on psychological resilience and quality of life in patients with type 2 diabetes showed that patients experienced a significant decrease in blood glucose levels and depression scores and a significant increase in psychological resilience and quality of life after the intervention.^22^ Another study noted that through a study based on the PMT-IMB construct as a structural model for explaining, predicting, and intervening in weight management behaviors during pregnancy, it was found that information about pregnancy weight management had a direct positive effect on weight management behaviors during pregnancy.^17^ The combination of PMT-IMB theory emphasizes the individual's intrinsic motivation and self-efficacy in behavior, aiming to stimulate the motivation for individuals to engage in healthy behaviors, thereby fully leveraging the subjective initiative of healthy behaviors. This approach seeks to better interpret the behavior changes and mastery of preventive behavior techniques in breast cancer patients, and to implement personalized intervention plans for individuals. To date, however, there have been no studies utilizing the PMT-IMB theory to prevent postoperative lymphedema in patients with breast cancer. Therefore, this study conducts nursing interventions based on the PMT-IMB theory to verify whether this theoretical model can help patients establish motivation to prevent lymphedema, acquire knowledge about lymphedema prevention, and take preventive measures.

## Methods

### Participants

In this study, all subjects were selected from the Department of Breast Surgery, Affiliated Hospital of Jiangnan University, Wuxi, Jiangsu, China, and patients with breast cancer treated between December 2022 and June 2023 were selected as study subjects. The study was conducted using a double-blind method, with participants randomly assigned to the treatment group through a computer system to ensure that information distribution would not be revealed in advance. Subjects were randomly assigned to the control group and the intervention group, usually by an independent person who was unaware of the purpose and recruitment process of the experiment. The allocation process was based on a computer-generated random number table to ensure fairness to the researchers. The inclusion criteria were as follows: (1) Patients with unilateral breast cancer who underwent modified radical mastectomy with sequential radiotherapy for patients with breast cancer. (2) Age greater than 18 years old. (3) Consciousness and no language communication disorder. (4) No lymphedema occurred when the patients were enrolled. (5) Informed consent and voluntary participation. Exclusion criteria: (1) Combination of other serious diseases, such as heart failure. (2) Patients with bilateral breast cancer, recurrence and other organ metastasis. (3) Open or infected wounds on the upper limbs. All study subjects were selected based on strict inclusion and exclusion criteria.

### Sample size calculation

**Fig. 1:**
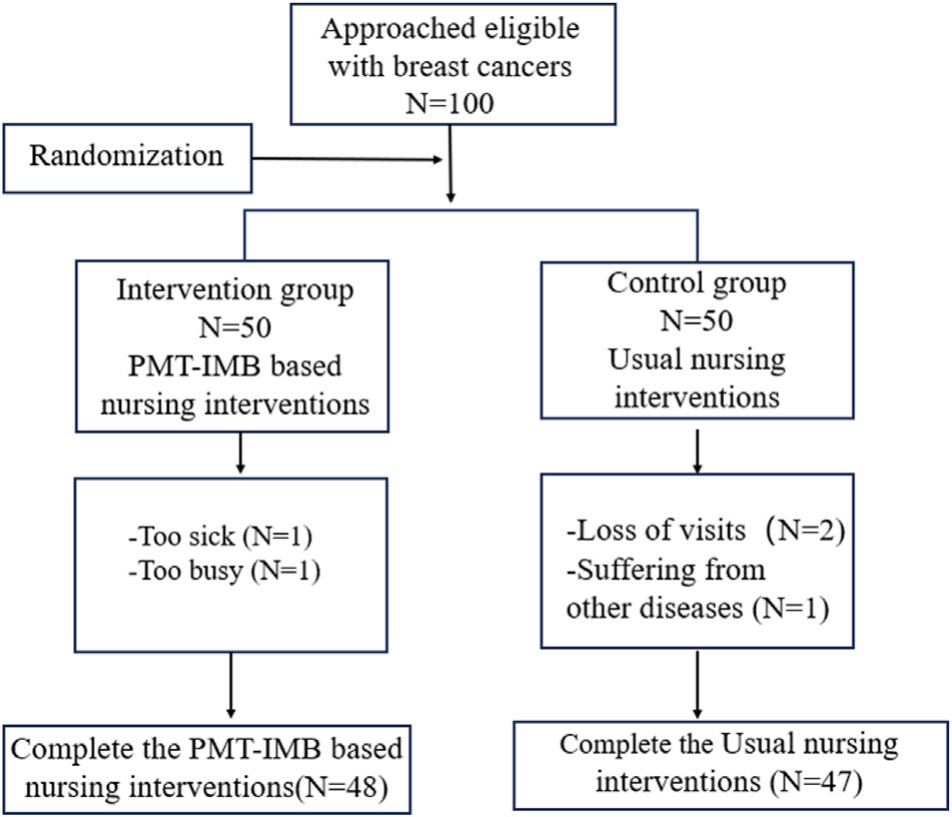
Flowchart of the study. PMT-IMB, Protection Motivation Theory and Information-Motivation-Behavioral Skills Model.

Reviewing the literature and referring to previous studies,^23^ the incidence of lymphedema was used as an evaluation index, which was calculated by Tests for Two Proportions of PASS 15.0, with power set to 0.90 and Alpha to 0.05, and the total sample size was calculated to be 80 cases, 40 cases in each of the two groups, and taking into account the 20% loss of visit rate in the present study, the total sample size was finally confirmed to be 100 cases, 50 cases in each of the two groups. A total of two patients in the intervention group withdrew during the intervention; a total of three patients in the control group withdrew from the study; 95 patients were eventually enrolled in the study, 48 in the intervention group and 47 in the control group. The study flow of participants into the program is shown in Fig. 1.

### Measurements

(1) Questionnaire for general information

The general information questionnaire was designed on its own to collect basic demographic information about the participants and contained two main sections. The first part was general information, i.e., age, race, contact information, marital status, education level, BMI, and occupational status. The second part of information mainly included the name of the surgery, pathologic stage, lymph node metastasis, and treatment modality.

(2) Lymphedema Risk-Reduction Behavior Checklist

The Breast Cancer Lymphedema Risk-Reduction Behavior Checklist (LRRB) was compiled by FU and other research scholars through the U.S.^24^, ^25^, ^26^ National Lymphedema Network and is mainly used to evaluate the ability of breast cancer patients to perform BCRL prevention,^9^^,^^24^^,^^27^ which includes four items, including skin care, lifestyle, avoidance of upper extremity compression and promotion of lymphedema, and other four items, using a 0–3 scale. The total score was 51 points. Higher scores represent higher patient competence and adherence to lymphedema prevention. The Cronbach's alpha coefficient for this scale was 0.830.

(3) Disabilities of the Arm, Shoulder and Hand

The Disabilities of the Arm, Shoulder and Hand (DASH) scale was developed by Beaton et al., in 2005 to assess the functional status of the patient's limb on the operated side and to respond to the patient's subjective feelings about the limb on the operated side.^28^ The scale contains a total of 11 items that focus on social functioning, physical activity, and the study participants' assessment of their own upper extremity symptoms. In the section assessing the patient's daily functioning, each item is divided into five levels based on the degree of difficulty the patient has in completing the task: 1 for no difficulty, 2 for slight difficulty, 3 for difficulty but achievable, 4 for difficult to do, and 5 for impossible to do. Upper extremity symptoms were also scored according to the severity of the symptoms: 1 for no symptoms, 2 for mild symptoms, 3 for moderate symptoms, 4 for severe symptoms, and 5 for extreme symptoms, and the DASH score was calculated as DASH ​= ​[(patient's score/number of responding entries)−1] ​× ​25, with a range of 0–100, and the higher the score, the more severe the upper extremity dysfunction of the patient. Its scale Cronbach's ɑ coefficient was 0.911, and the degree of stability was 0.882, which was good.

(4) Upper limb circumference measurement

The scale is used to detect the occurrence and severity of lymphedema in patients. In this study, the researcher issued a soft tape measure to the patients for measuring the circumference of the upper limbs. The main areas of measurement included the circumference of the tiger's mouth of both hands, the transverse carpal tunnel, 10 ​cm below the transverse elbow, 10 ​cm above the transverse elbow, and 20 ​cm above the transverse elbow, with a gap of ≥ 2.0 ​cm in any one of these areas being diagnosed as lymphedema.^29^ And according to the American Physical Therapy Association, which categorizes lymphedema as mild, moderate, or severe, for patients diagnosed with lymphedema, if the difference in circumference between the two upper extremities is less than 3.0 ​cm it is considered mild edema, a difference between 3.0 and 5.0 ​cm is considered moderate, and greater than 5.0 ​cm is considered severe edema.

(5) Self-Lymphatic Drainage Adherence Scale

According to the Summary of Evidence for Prevention of Upper Extremity Lymphedema in patients with breast cancer, for those who performed self-lymphatic drainage threetimes a day for a duration of at least 10 ​min each time to achieve the total weekly exercise time recommended for prevention (210 ​min/week) were considered to have good self-lymphatic drainage compliance, and vice versa, they were considered to have poor self-lymphatic drainage compliance.^30^

### Data analysis

The SPSS 27.0 program was used to process and analyze the data, and *P* ​< ​0.05 indicated that the difference was statistically significant. For count data, it was described by frequency and percentage, and Fisher's exact test and chi-square test were used to compare the differences between the groups; Measurements were described using mean ​± ​standard deviation, and comparisons between groups were analyzed using the independent samples *t* test and rank sum test. The preventive behavior scores and upper limb dysfunction scores of the two study groups were analyzed and compared using independent samples *t* test; Trends in the emergence of preventive behaviors, functional status, and patient circumference over time were compared pre-intervention, 1 month post-intervention, 3 months post-intervention, and 6 months post-intervention by applying repeated-measures ANOVA, and analyzing between-group and point-in-time and between-group interaction effects; The incidence of lymphedema was analyzed using the continuous corrected chi-square test and Fisher's exact test. Longitudinal changes in adherence to self-lymphatic drainage in both groups were analyzed using the generalized estimating equation test.

### Intervention procedure

(1) Formation of the PMT-IMB Research Team

The members of the PMT-IMB research team, based on PMT-IMB nursing intervention for the prevention of lymphedema, include one graduate advisor who supervised and guided this research plan. One physician from the breast surgery department and one physician from the radiotherapy department provided training to the team members on patients with breast cancer and the prevention of lymphedema and also address any related questions from the patients. Two patients with breast cancer radiotherapy nurses assisted the researchers in implementing specific intervention measures. One lymphedema care specialist provided patients with breast cancer with knowledge and technical guidance on lymphedema prevention and answered their questions. One nursing graduate student, involved in the formulation, implementation, data collection, and processing of the entire intervention plan.

(2) Compile a health manual for the prevention of lymphedema

Based on the evidence summary for the prevention of lymphedema and a literature review, a “Manual for Preventing Postoperative Upper Limb Lymphedema” was developed for the subjects of this study, and it was refined and modified following discussions by the research team. The main sections include: ① What is lymphedema; ② What are the risk factors for lymphedema; ③ the harms of lymphedema; ④ How to prevent lymphedema; ⑤ How to identify.

(3) Establish a WeChat public account

The research team established a WeChat public account and used the platform to disseminate information, mainly covering three major sections: knowledge on preventing lymphedema, motivation support for prevention, and prevention behavior techniques. The content was reviewed by lymphedema care experts before being published, as detailed in Supplementary Table S1.

**Fig. 2:**
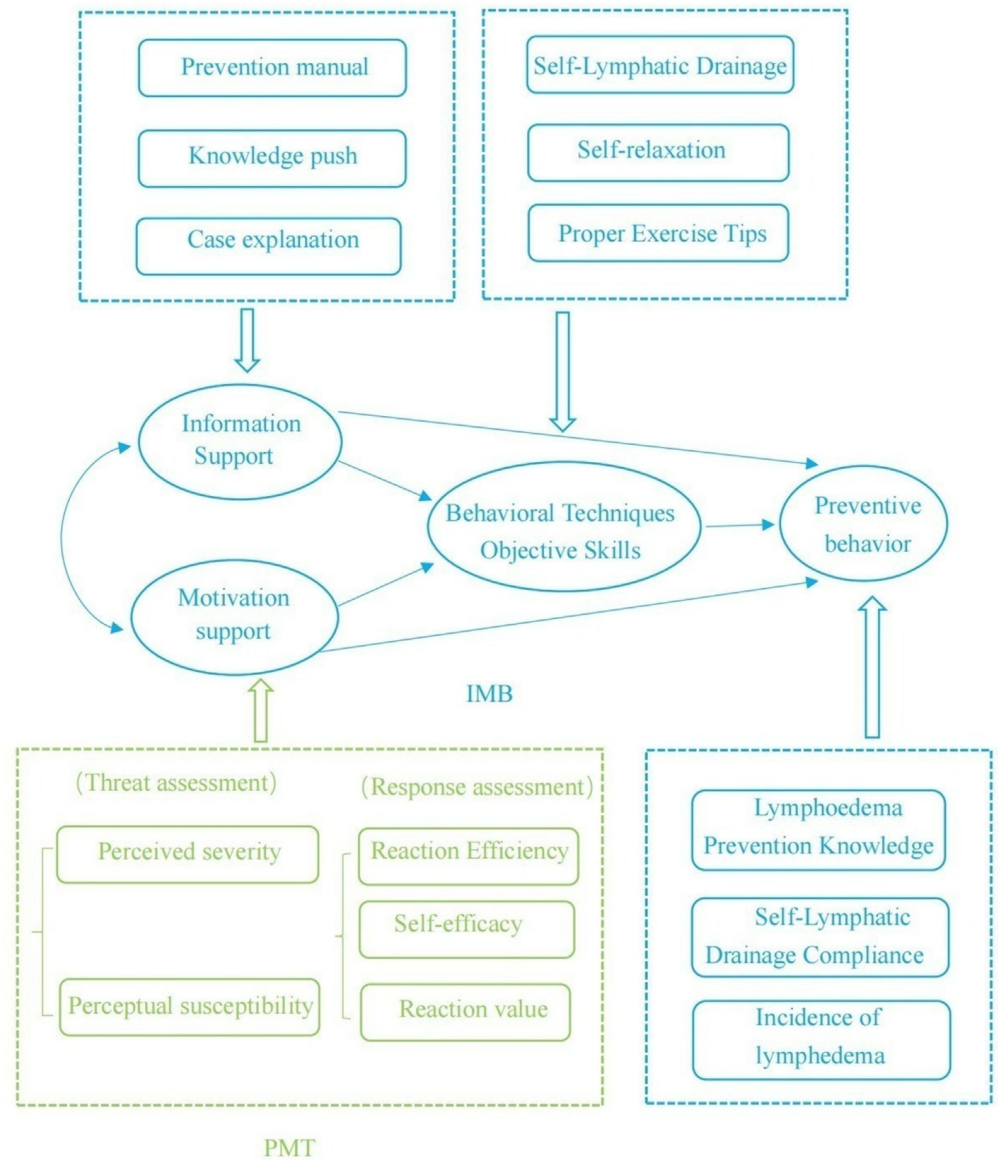
The PMT-IMB theoretical model diagram. PMT-IMB, Protection Motivation Theory and Information-Motivation-Behavioral Skills Model.

The researcher has read a lot of domestic and international literature to master the theoretical knowledge and nursing program for the prevention of lymphedema in patients with breast cancer, combined with the application of the PMT-IMB theory, and initially formed a nursing intervention program based on the PMT-IMB theory after discussion in the research group. The intervention program used in this study was developed based on the Protection Motivation Theory–Information-Motivation-Behavioral Skills Model.^18^^,^^31^ (The PMT-IMB theoretical model diagram is shown in Fig. 2). The content consists of three modules: information support, motivation support, and behavioral skill support. Considering the characteristics of the patient's disease, the length of hospitalization and the development of the Internet in the new era, a combination of online and offline methods was used in this study. (1) Information support intervention: to explain to patients with breast cancer about the causes of breast cancer disease, breast cancer surgical methods and complications, risk factors for the occurrence of BCRL, recognizing the early symptoms of lymphedema and how to effectively prevent the occurrence of lymphedema, and other related knowledge; (2) Motivational support: health care and family members accompany and encourage patients to enhance their perceived severity and susceptibility to the disease, enhance self-efficacy and response efficacy, so as to motivate patients to generate beliefs and motivation to change health behaviors; to assist patients in setting behavioral goals and carrying out regular preventive behaviors; (3) Behavioral skills: teaching patients with breast cancer to perform proper exercise, self-lymphatic drainage, and psychological adjustment skills, etc.

In this study, 10 representative experts were selected through purposive sampling to participate in the revision of this study protocol. We sent the first draft of the intervention protocol to the experts for review and adjusted the intervention protocol based on the suggestions given by the expert panelists (Supplementary Table S2 for detailed information on the experts' correspondence) and finally formed the specific study protocol (Supplementary Table S2 for the specific intervention protocol).

In addition, we report on the adverse events that occurred in this study and their management (Supplementary Table S3 for details).

### Follow up

After the end of the intervention, patients were followed up by telephone or WeChat platform, every two weeks from 13 to 17 weeks, and every four weeks from 17 to 24 weeks, with each follow-up visit lasting 20–30 minutes, and the follow-up visits to patients were as follows: (1) asking patients about their recent adherence to the preventive behaviors, helping patients analyze the positive impacts of the health behaviors, and affirming the health behaviors the patients made in their efforts, and encourage the patient to continue to maintain. (2) Ask the patient about the condition of the upper limbs and inform the patient of the importance of self-lymphatic drainage to prevent lymphedema; ask the patient about the problems and difficulties encountered by the patient and assist the patient in formulating solutions. (3) Keep abreast of the patient's psychological dynamics and give the patient support. Tell the patient to go to the hospital on time for follow-up, etc.

## Results

### Comparison of baseline data between the two groups of patients

In this study, data were finally collected from 95 patients with breast cancer, and all the study subjects were female. The average age of the control group (53.47 ​± ​11.34) and the average age of the intervention group (52.90 ​± ​8.13); the education level of junior high school education is more, and the proportion of the control group and the intervention group is 53.2% and 62.5%, respectively. By comparing the pre-intervention control group and intervention group in terms of age, BMI, marriage, occupational status, education level and tumor-node-metastasis (TNM) staging, the results showed that the differences were not statistically significant (*P* ​> ​0.05) (Table 1).

**Table 1:** Comparison of demographic data of the two groups of patients.

Items		Control group (*n* = 47)	Intervention group (*n* = 48)	*t/*χ^*2*^*/z*	*P*
Age (years), Mean ± SD		53.47 ​± ​11.34	52.90 ​± ​8.13	−0.283^a^	0.778
BMI (kg/m^2^), Mean ± SD		23.83 ​± ​3.04	24.00 ​± ​3.03	−0.179^a^	0.858
Marriage	Married	46 (97.9%)	47 (97.9%)	−0.015^b^	0.988
	Divorced	1 (2.1%)	1 (2.1%)		
Occupational status	Unemployed	13 (27.7%)	24 (50.0%)	5.001^b^	0.082
	Employed	15 (31.9%)	11 (22.9%)		
	Retired	19 (40.4%)	13 (27.1%)		
Education level	Elementary and below	7 (14.9%)	6 (12.5%)	1.921^b^	0.589
	Junior high school	25 (53.2%)	30 (62.5%)		
	High school/Middle school	7 (14.9%)	8 (16.7%)		
	College and above	8 (17.0%)	4 (8.3%)		
TNM stage	I	2 (4.3%)	0 (0.0%)	2.992^b^	0.224
	II	26 (55.3%)	23 (47.9%)		
	III	19 (40.4%)	25 (52.1%)		
	IV	0 (0.0%)	0 (0.0%)		
Lymphatic node transfer	With	30 (63.8%)	33 (68.8%)	−0.505^c^	0.614
	Without	17 (36.2%)	15 (31.3%)		
Chemotherapy	Yes	46 (97.9%)	47 (97.9%)	−0.015^b^	0.988
	No	1 (2.1%)	1 (2.1%)		
Surgical location	Left	21 (44.7%)	28 (58.3%)	−1.3245^c^	0.185
	Right	26 (55.3%)	20 (41.7%)		

BMI, body mass index; TNM, tumor-node-metastasis. a. two independent samples *t* test; b. chi-square test; c. Mann–Whitney *U* rank sum test.

### Lymphedema prevention behavior scores in both groups before and after intervention

(1) Comparison of the total score of lymphedema prevention behaviors between the two groups before and after intervention

By analyzing and comparing the status of lymphedema preventive behavior scores of the two groups of patients, it can be seen that before the intervention: the intervention group's preventive behavior score was (7.08 ​± ​2.28), compared with that of the control group (7.26 ​± ​2.18), there was no statistically significant difference (*t* ​= ​0.376, *P* ​> ​0.05). 1 month after the intervention: the preventive behavior score of the intervention group was (39.12 ​± ​3.84), which was statistically significantly different from the control group (21.70 ​± ​4.85) (*t* ​= ​−19.398, *P* ​< ​0.001); 3 months after the intervention: the preventive behavior score of the intervention group was (37.81 ​± ​6.07), which was statistically significantly different from the control group (20.82 ​± ​4.67) (*t* ​= ​−15.227, *P* ​< ​0.001); 6 months after the intervention: the preventive behavior score of the intervention group was (35.92 ​± ​7.45), which was statistically significantly different from the control group (18.45 ​± ​4.80) (*t* ​= ​−13.553, *P* ​< ​0.001) (Table 2). The results of the repeated measures ANOVA showed (Table 2) that the data did not satisfy the hypothesis test for sphericity (*P* ​< ​0.05) as indicated by the Mauchly's test for sphericity, and as indicated by the Greenhouse-Geisser correction. (1) Time effect: the total score of preventive behaviors had statistically significant differences between the two groups of patients at different time points before and after the intervention (*F*_time_ ​= ​914.626, *P* ​< ​0.001); (2) Group effect: there was a statistically significant difference in the total score of preventive behaviors between the two groups of the intervention group and the control group (*F*_group_ ​= ​458.378, *P* ​< ​0.001). (3) Interaction effect: the total score of preventive behaviors of patients in the two groups changed with the change of measurement time points, and the difference was statistically significant (*F*_interaction_ ​= ​136.351, *P* ​< ​0.001).

(2) Comparison of the scores of the dimensions of lymphedema preventive behaviors between the two groups of patients before and after the intervention

**Table 2:** Comparison of total knowledge of lymphedema prevention scores between the two groups before and after intervention.

Groups	E_0_	E_1_	E_3_	E_6_	*F*_*time*_^a^	*F*_*group*_^a^	*F*_*interaction*_^a^
Control group (*n* = 47)	7.26 ​± ​2.18	21.70 ​± ​4.85	20.82 ​± ​4.67	18.45 ​± ​4.80	914.626	458.378	136.351
Intervention group (*n* = 48)	7.08 ​± ​2.28	39.12 ​± ​3.84	37.81 ​± ​6.07	35.92 ​± ​7.45			
*t*	0.376	−19.398	−15.227	−13.553			
*P*	0.708	< 0.001	< 0.001	< 0.001	< 0.001	< 0.001	< 0.001

E_0_: pre-intervention; E_1_: post-intervention month 1; E_3_: post-intervention month 3; E_6_: post-intervention month 6.

a Greenhouse-Geisser correction; *t*: Independent samples *t* test.

By analyzing the comparison of the scores of the dimensions of lymphedema preventive behaviors between the two groups of patients before and after the intervention, the results showed that there was no statistically significant difference in the scores of the dimensions of avoiding injury situations of the affected limbs, skin care, lifestyle and promoting lymphatic fluid reflux before the intervention (*P* ​> ​0.05).

The scores of all dimensions in the intervention group were significantly higher than those of the control group at 1 month post-intervention, 3 months post-intervention and 6 months post-intervention, and the difference was statistically significant (*P* ​< ​0.05) (Table 3). The data did not satisfy the test of the assumption of sphericity (*P* ​< ​0.05) by repeated-measures ANOVA of the knowledge scores of the two groups of patients on avoiding injuries to the affected limbs, as shown by the Greenhouse-Geisser corrected results: There was a between-group difference in the avoidance of injury to the affected limb score in the preventive behavior scores between the two groups, which was statistically significant (*F*_group_ ​= ​338.870, *P* ​< ​0.001); there was also a statistically significant difference in the interaction effect and the time effect (*F*_interaction_ ​= ​69.308, *F*_time_ ​= ​168.976, both *P* ​< ​0.001) (Table 3).

**Table 3:** Scores on the dimensions of knowledge of lymphedema prevention in both groups of patients.

Item	Group	E_0_	E_1_	E_3_	E_6_	*F*_*time*_^a^	*F*_*group*_^a^	*F*_*interaction*_^a^
Avoid injury to the affected limb	Control group	4.30 ​± ​1.30	6.17 ​± ​1.54	5.43 ​± ​1.74	5.47 ​± ​2.32	168.976	338.870	69.308
	Intervention group	4.69 ​± ​1.45	12.54 ​± ​1.95	11.25 ​± ​2.86	10.79 ​± ​2.90			
	*t*	−1.380	−17.688	−12.010	−9.861			
	*P*	0.171	< 0.001	< 0.001	< 0.001	< 0.001	< 0.001	< 0.001
Skin care	Control group	0.21 ​± ​0.62	4.98 ​± ​1.66	4.83 ​± ​1.97	4.40 ​± ​1.90	742.428	42.878	125.904
	Intervention group	0.17 ​± ​0.78	8.00 ​± ​0.72	7.60 ​± ​11.38	6.94 ​± ​2.82			
	*t*	0.318	−11.471	−7.934	−5.878			
	*P*	0.752	<0.001	<0.001	<0.001	<0.001	<0.001	<0.001
Lifestyle	Control group	1.43 ​± ​1.35	5.11 ​± ​1.67	5.09 ​± ​1.98	4.38 ​± ​2.00	259.748	216.855	41.694
	Intervention group	1.15 ​± ​1.29	9.88 ​± ​1.21	8.58 ​± ​2.06	8.67 ​± ​2.52			
	*t*	1.034	−16.019	−8.441	−9.172			
	*P*	0.304	< 0.001	< 0.001	< 0.001	< 0.001	< 0.001	< 0.001
Promotes the return of lymphatic fluid	Control group	1.34 ​± ​0.92	5.49 ​± ​2.23	5.40 ​± ​1.93	4.15 ​± ​1.59	359.602	188.059	54.639
	Intervention group	1.04 ​± ​1.03	8.65 ​± ​2.57	10.31 ​± ​2.75	9.27 ​± ​3.36			
	*t*	1.493	−6.391	−10.078	−9.524			
	*P*	0.139	< 0.001	< 0.001	< 0.001	< 0.001	< 0.001	< 0.001

E_0_: pre-intervention; E_1_: post-intervention month 1; E_3_: post-intervention month 3; E_6_: post-intervention month 6.

a Greenhouse-Geisser correction; *t*: Independent samples *t* test.

The data did not satisfy the hypothesis test for sphericity (*P* ​< ​0.05) by repeated measures ANOVA on the skin care knowledge scores of the two groups of patients, which was corrected by Greenhouse-Geisser to show that there was a between-group difference in the skin care scores of the two groups that was statistically significant (*F*_group_ ​= ​42.878, *P* ​< ​0.001), and that the effect in the interaction and the time effect were statistically significant (*F*_interaction_ ​= ​125.904, *F*_time_ ​= ​742.428, both *P* ​< ​0.001) (Table 3).

The data did not satisfy the test of the assumption of sphericity by repeated measures ANOVA on the lifestyle knowledge scores of the two groups (*P* ​< ​0.05), and the results of the Greenhouse-Geisser correction showed that there was a significant between-group difference in the lifestyle scores of the two groups, which was statistically significant in all effects (*F*_group_ ​= ​216.855, *F*_interaction_ ​= ​41.694, *F*_time_ ​= ​259.748, all *P* ​< ​0.001) (Table 3).

The data did not satisfy the test of the assumption of sphericity (*P* ​< ​0.05) by repeated measures ANOVA on the knowledge score of promoting lymphatic reflux in the two groups, which was corrected for the Greenhouse-Geisser correction, showed that there was a significant between-group difference in the knowledge score of promoting lymphatic reflux in the two groups, and that it was statistically significant for each effect (*F*_group_ ​= ​188.059, *F*_interaction_ ​= ​54.639, *F*_time_ ​= ​359.602, all *P* ​< ​0.001) (Table 3).

### Upper limb dysfunction scores in both groups before and after intervention

By comparing the upper limb dysfunction scores of the two groups of patients before and after the intervention, the results indicated that: before the intervention: the upper limb dysfunction scores of the intervention group and the control group were (24.19 ​± ​5.50) and (22.25 ​± ​6.70), respectively, and there was no statistically significant difference between the two groups compared to the two groups (*t* ​= ​1.547, *P* ​> ​0.05); 1 month post-intervention: the upper limb dysfunction scores in the experimental and control groups were (21.78 ​± ​5.64) and (23.88 ​± ​7.17), respectively, and there was no statistically significant difference between the two groups compared to each other (*t* ​= ​−1.594, *P* ​> ​0.05); 3 months after the intervention: the upper limb dysfunction scores of the experimental and control groups were (18.61 ​± ​6.34) and (25.87 ​± ​10.12), respectively, with a statistically significant difference between the two groups (*t* ​= ​−4.180, *P* ​< ​0.001); 6 months after the intervention: the upper limb dysfunction score of the intervention group was (16.52 ​± ​9.61) lower than that of the control group (26.00 ​± ​16.04), with a statistically significant difference between the two groups compared to each other (*t* ​= ​−3.830, *P* ​< ​0.001) (Table 4).

**Table 4:** Upper limb dysfunction scores of the two groups of patients.

Group	E_0_	E_1_	E_3_	E_6_	*F*_*time*_^a^	*F*_*group*_^a^	*F*_*interaction*_^a^
Control group	22.25 ​± ​6.70	23.88 ​± ​7.17	25.87 ​± ​10.12	26.00 ​± ​16.04	0.602	8.483	12.077
Intervention group	24.19 ​± ​5.50	21.78 ​± ​5.64	18.61 ​± ​6.34	16.52 ​± ​9.61			
*t*	1.547	−1.594	−4.180	−3.830			
*P*	0.125	0.114	< 0.001	< 0.001	0.615	0.004	< 0.001

E_0_: pre-intervention; E_1_: post-intervention month 1; E_3_: post-intervention month 3; E_6_: post-intervention month 6.

a Greenhouse-Geisser correction; *t*: Independent samples *t* test.

By using repeated measures ANOVA results showed that (1) time effect: upper limb dysfunction scores were not statistically different among the different measurement time points (*F*_time_ ​= ​0.602, *P* ​> ​0.05); (2) grouping effect: upper limb dysfunction scores were statistically different among different groups (*F*_group_ ​= ​8.483, *P* ​< ​0.05); (3) interaction effect was found for the further analysis of the data for time point and group interaction, again a statistically significant difference was found (*F*_interaction_ ​= ​12.077, *P* ​< ​0.001) (Table 4).

### Comparison of the effect of prevention of lymphedema in two groups of patients before and after intervention

(1) Comparison of the incidence of lymphedema between the two groups of patients

The results of the study showed that the incidence of limb lymphedema in the two groups was not statistically significant at 1 month and 3 months after intervention (*P* ​> ​0.05), and the incidence of limb lymphedema in the intervention group and the control group at 6 months after intervention was 6.3% (3 cases) and 27.7% (13 cases), respectively, with three cases in the intervention group suffering from mild edema, and 10 cases in the control group suffering from mild, two cases of moderate, and 1 case of severe edema, with a statistical difference (*P* ​< ​0.05). And 1 case of severe edema, and the incidence of lymphedema in the affected limbs of patients in the two groups was statistically different (*P* ​< ​0.05) (Table 5).

(2) Comparison of the circumference of the affected limb before and after the intervention in the two groups of patients

**Table 5:** Comparison of the incidence of lymphedema between the two groups of patients.

Time	Group	No edema	Mild edema	Moderate edema	Severe edema	χ^*2*^	*P*
**E**_**1**_	Control group (*n* = 47)	46 (97.90)	1 (2.10)	0 (0.00)	0 (0.00)	1.032	0.495
	Intervention group (*n* = 48)	48 (100.00)	0 (0.00)	0 (0.00)	0 (0.00)		
**E**_**3**_	Control group (*n* = 47)	41 (87.20)	4 (8.50)	2 (4.30)	0 (0.00)	3.674	0.089
	Intervention group (*n* = 48)	47 (97.90)	1 (2.10)	0 (0.00)	0 (0.00)		
**E**_**6**_	Control group (*n* = 47)	34 (72.30)	10 (21.30)	2 (4.30)	1 (2.10)	7.764	0.017
	Intervention group (*n* = 48)	45 (93.80)	3 (6.30)	0 (0.00)	0 (0.00)		

E_1_: Month 1 post-intervention; E_3_: Month 3 post-intervention; E_6_: Month 6 post-intervention.

A simple effects analysis using repeated measures ANOVA showed that the circumferences of the tiger's mouth, the transverse carpal tunnel, the 10 ​cm below the transverse elbow, the 10 ​cm above the transverse elbow, and the 20 ​cm above the transverse elbow of the affected limb in the control group increased progressively over time; The circumference of each part of the affected limb in both groups was not statistically significant before intervention, 1 month after intervention and 3 months after intervention (*P* ​> ​0.05), while there was a statistically significant difference between the tiger's mouth, transverse carpal tunnel, 10 ​cm below the transverse elbow, 10 ​cm above the transverse elbow and 20 ​cm above the transverse elbow at 6 months after intervention (*P* ​< ​0.05) (Table 6).

**Table 6:** Comparison of the circumference of the affected limb in the two groups of patients.

Item	Group	E_0_	E_1_	E_3_	E_6_
The web between the thumb and forefinger of the hand	Control group	18.19 ​± ​1.65	18.30 ​± ​1.67	18.61 ​± ​1.81	19.07 ​± ​1.98
	Intervention group	18.08 ​± ​1.36	18.10 ​± ​1.31	18.17 ​± ​1.26	18.33 ​± ​1.38
	*F*	0.122	0.397	1.888	4.499
	*P*	0.728	0.53	0.173	0.037
Transverse stripe of the wrist	Control group	15.34 ​± ​1.41	15.47 ​± ​1.38	15.67 ​± ​1.53	16.28 ​± ​1.84
	Intervention group	15.25 ​± ​1.19	15.30 ​± ​1.15	15.35 ​± ​1.27	15.45 ​± ​1.39
	*F*	0.114	0.406	1.205	6.177
	*P*	0.736	0.526	0.275	0.015
10 ​cm below the transverse elbow stripe	Control group	19.67 ​± ​2.39	19.72 ​± ​2.39	19.84 ​± ​2.46	20.70 ​± ​2.56
	Intervention group	19.45 ​± ​2.12	19.47 ​± ​2.11	19.49 ​± ​2.12	19.55 ​± ​2.15
	*F*	0.209	0.303	0.555	5.629
	*P*	0.649	0.583	0.458	0.020
10 ​cm above the transverse line of the elbow	Control group	28.48 ​± ​3.06	28.52 ​± ​3.04	28.61 ​± ​3.11	29.76 ​± ​3.34
	Intervention group	28.30 ​± ​2.98	28.33 ​± ​2.94	28.39 ​± ​2.88	28.43 ​± ​2.89
	*F*	0.081	0.094	0.117	4.230
	*P*	0.776	0.760	0.733	0.043
20 ​cm above the transverse line of the elbow	Control group	33.68 ​± ​3.59	33.78 ​± ​3.55	33.86 ​± ​3.59	34.92 ​± ​3.82
	Intervention group	33.50 ​± ​2.92	33.60 ​± ​2.86	33.62 ​± ​2.87	33.54 ​± ​2.81
	*F*	0.073	0.068	0.137	3.993
	*P*	0.788	0.795	0.712	0.049

E_0_: pre-intervention; E_1_: post-intervention month 1; E_3_: post-intervention month 3; E_6_: post-intervention month 6.

*F*: Fisher's exact test.

### Comparison of adherence to self-lymphatic drainage in two groups of patients

Intergroup comparison of self-lymphatic drainage compliance between the two groups of patients by chi-square test showed that self-lymphatic drainage compliance in the intervention group was higher than that in the control group at 1 month after the intervention, 3 months after the intervention, and 6 months after the intervention, and there was a statistically significant difference between the two groups (*P* ​< ​0.05). The categorical variable self-lymphatic drainage adherence was further analyzed by generalized estimating equations, and the results of the model fitting showed statistically significant differences in the group and time effects of self-lymphatic drainage adherence (Wald χ^2^ ​= ​17.352, *P* ​< ​0.001; Wald χ^2^ ​= ​10.787, *P* ​< ​0.05) (Table 7).

**Table 7:** Comparison of adherence to self-lymphatic drainage in the two groups after intervention.

	E_1_		E_3_		E_6_		Group effect	Time effect
Group	Good	Bad	Good	Bad	Good	Bad	Wald χ^2^	Wald χ^2^
Control group (*n* = 47)	34 (72.3)	13 (27.7)	29 (61.7)	18 (38.3)	24 (51.1)	23 (48.9)	17.352	10.787
Intervention group (*n* = 48)	45 (93.8)	3 (6.2)	43 (89.6)	5 (10.4)	40 (83.3)	8 (16.7)		
χ^2^	7.772		10.061		11.249			
*P*	0.005		0.002		< 0.001		< 0.001	0.005

E_1_: post-intervention month 1; E_3_: post-intervention month 3; E_6_: post-intervention month 6.

## Discussion

Lymphedema associated with breast cancer remains incurable in clinical practice. The condition is characterized by symptoms such as swelling, pressure and pain, and heaviness in the armpits and upper limbs of the affected side of the patient. In severe cases, it can result in limb function impairment, thereby affecting the patient's daily life activities and working ability. The swelling of limbs may also lead to a decline in the patient's self-esteem, potentially resulting in negative emotions. Furthermore, the accumulation of lymphatic fluid provides a breeding environment for bacteria, thereby increasing the risk of infection. The long-term existence of lymphedema may lead to further deterioration and dysfunction of the lymphatic system, which has a significant impact on the quality of life of patients with breast cancer. Therefore, the prevention of breast cancer-related lymphedema is of particular importance. Based on the PMT-IMB intervention protocol as a guide, this study proposes a new nursing model for the prevention of lymphedema in patients with breast cancer, aiming to increase patients' knowledge and behaviors of lymphedema prevention, reduce the incidence of lymphedema, and thus improve patients' quality of life. However, the present study was not subject to external validation and its efficacy was only verified in a single center. Furthermore, the total sample size was relatively small and under-representative. Consequently, the results of the study should be viewed with caution and the sample size should be expanded in future to carry out a multicenter, large-sample study on the prevention of lymphedema in patients with breast cancer, so as to increase the representativeness of the sample. Furthermore, the majority of patients received the intervention during the autumn and winter months, and the impact of climate on the occurrence of lymphedema remains to be elucidated. Consequently, future studies should explore this factor in depth to provide further confirmation of the effectiveness of the intervention program. Notwithstanding these uncertainties, the present study remains pertinent in terms of future directions. A 12-week nursing intervention, grounded in the PMT-IMB intervention protocol, has yielded relatively effective nursing outcomes. The subsequent discussion of lymphedema prevention strategies for breast cancer patients is to be informed by these findings.

### PMT-IMB-based intervention program enhances patients' lymphedema prevention behaviors

Patients undergoing axillary lymph node dissection and radiotherapy are more prone to developing lymphedema; therefore, patients in this study should pay special attention to the occurrence of lymphedema. Many studies have pointed out that effective behavioral interventions can reduce the risk of lymphedema in breast cancer patients, but the patients' ability to execute preventive measures is reduced, and the effect is not significant.^32^ There is a strong consensus emphasizing that providing patients with comprehensive education on lymphedema. After the nursing intervention in this study, the total score of lymphedema preventive behavior of the patients in the intervention group was (7.08 ​± ​2.28) before the intervention and improved to (35.92 ​± ​7.45) after the 6-month intervention, which indicates that patient-centeredness, the provision of effective preventive information, and the enhancement of the patient's motivation for prevention lead to the ability of patients to develop effective preventive behaviors.

During the hospitalization period, the researchers provided face-to-face oral education to the patients and distributed preventive pamphlets, informing them about the important risk factors for the occurrence of lymphedema, the harm it brings, and the importance of preventing lymphedema, thereby increasing the patients' awareness of lymphedema prevention. After the patient is discharged, we continuously provide preventive knowledge through WeChat public accounts and remind patients to study. For any questions raised by the patients, we promptly provide answers and guidance. This is consistent with research by scholars such as Bili Gao.^33^ By providing health education to breast cancer patients and offering preventive knowledge and guidance, we can greatly enhance patients' understanding of the disease and their proactive engagement.

### PMT-IMB-based intervention program improves patients' upper limb functional status

Upper limb dysfunction may cause patients to experience pain in the affected limb, restricted joint movement, and sensory abnormalities. According to most studies, breast cancer surgery and radiotherapy may damage the blood vessels and nerves in the patient's armpit and chest wall, leading to the formation of local scar tissue. This, in turn, restricts the range of motion of the joints, reduces the patient's physical activity, and exacerbates the functional impairment of the affected limb. The study also pointed out that early use of manual lymphatic drainage and proper functional exercises can effectively improve the patient's upper limb function.^34^ However, some patients with breast cancer reduce or even limit the activity of the affected limb due to concerns about complications, and treatments such as axillary clearance and radiotherapy can cause pain in the affected limb, leading to restricted movement of the affected upper limb, thereby increasing the risk of lymphedema and creating a serious adverse cycle. This study, using the Upper Limb Function Simple Questionnaire, found no statistical difference in upper limb function scores between the experimental and control groups one-month post-intervention. This may be due to the short follow-up period, resulting in no significant differences in the outcomes between the two groups of patients. However, at three and 6 months post-intervention, the upper limb function scores of the intervention group patients were significantly lower than those of the control group, with a statistically significant difference between the two groups. Similar to the study by Yu Xiao et al.,^35^ conducting health education effectively improved patients' adherence to functional exercises, enhanced their upper limb function, and promoted the rehabilitation of their upper limb function. The intervention group, through the PMT-IMB-based intervention program, can exercise and perform self-lymphatic drainage more accurately and promptly, gradually improving the patients' self-care abilities. At the same time, by combining online and offline intervention modes, the program further enhances the patients' health behaviors, thereby reducing the degree of upper limb dysfunction.

### PMT-IMB-based intervention program reduces the incidence of lymphedema

Lymphedema is one of the common complications after breast cancer surgery. Its possible cause is the blockage of lymphatic drainage pathways due to axillary lymph node dissection, leading to the accumulation of protein-rich lymph fluid in the tissue interstices, resulting in chronic tissue edema. Currently, once it occurs clinically, it cannot be cured, so patients need to have knowledge and skills to prevent lymphedema.^36^ The results of this study indicate that the incidence of lymphedema at various stages after intervention in the intervention group was lower than that in the control group, and there was a significant statistical difference in the incidence of lymphedema between the two groups six months after enrollment. This is similar to the research results of scholars such as Ayse Cal.^37^ This study emphasizes the importance of information, motivation, and behavioral skills through an intervention program based on the PMT-IMB theoretical framework. Therefore, based on theoretical nursing education interventions, it is possible to reduce the incidence of lymphedema. However, since the intervention period is relatively short, we should view the intervention effects with caution.

### PMT-IMB-based intervention program improves patients' affected limb swelling

The results of this study show that after the intervention, the circumferences of the five measurement sites in the intervention group were all smaller than those in the control group. This indicates that the PMT-IMB-based nursing intervention program is superior to the control group. This may be because the patients in the control group had weaker knowledge retention and motivation compared to those in the intervention group, and there was a significant difference in preventive techniques. The patients in the intervention group possessed extensive knowledge about preventing lymphedema. However, the preventive behavior motivation of a small number of patients still needs improvement. Therefore, it reminds us that in future nursing interventions, we should strengthen information education, motivation support, and behavior guidance for patients, while also increasing the frequency of follow-ups. Moreover, six months after the intervention, there were statistically significant differences in all five sites between the two groups of patients, similar to the study by Gizmo and other scholars,^38^ which reduced the risk of lymphedema and the degree of swelling in the affected limbs through continuous health education. Based on information, motivation, and behavioral skills, improve patients' mastery of behavioral skills, enabling them to use more scientific and professional methods to prevent and manage the occurrence of edema. The team also provides encouragement and support to the patients, fully mobilizing their enthusiasm and enhancing their motivation and belief in preventing or reducing edema.

### PMT-IMB-based intervention program enhances patients' self-adherence to lymphatic drainage

Lymphatic drainage is an effective preventive method for preventing lymphedema in the upper limbs caused by breast cancer. Its mechanism of action involves massaging the superficial lymphatics of the upper limbs, which can to some extent increase the openness of lymphatic pathways; by applying pressure from the distal end towards the proximal end, it can help the accumulated lymph fluid in the body re-enter the bloodstream, thereby promoting the acceleration of lymphatic flow.^39^ Moreover, self-lymphatic drainage not only increases the flow rate of lymphatic drainage fluid but also reduces sympathetic nervous activity, enhances parasympathetic responses, thereby promoting hydrolysis within the body to reduce local swelling.^40^

The results of this study on patients' adherence to self-lymphatic drainage through the PMT-IMB-based intervention program showed that the intervention group showed statistically significant differences in self-lymphatic drainage adherence compared with the control group at 1 month, 3 months, and 6 months after the intervention. This is because this study, by combining two theories, has formulated a nursing intervention plan that greatly enhances patients' motivation for preventive behavior, thereby increasing their adherence to self-lymphatic drainage. This is consistent with previous research findings, where Zofia et al. enhanced handwashing compliance during the COVID-19 pandemic through an intervention program based on the PMT theory.^41^ In addition, Molly and others improved medication adherence behavior in kidney transplant patients through the IMB theory.^42^ This indicates that by combining the two theories and formulating nursing intervention plans based on them, patients' motivation for preventive behavior can be significantly enhanced. This includes increasing patients' perceived susceptibility, perceived severity, self-efficacy, and response efficacy, thereby increasing patients' self-lymphatic drainage adherence and greatly improving their health behavior.

## Conclusions

The nursing intervention program based on PMT-IMB in this study can effectively reduce the incidence of lymphedema and the degree of swelling of the affected limbs, improve upper limb dysfunction, and reduce its impact on daily life. At the same time, the intervention can effectively improve the ability of preventive behaviors of patients with breast cancer and promote the generation of preventive behaviors. In addition, it can enhance the generation of healthy behaviors in patients with breast cancer and improve the patients' adherence to self-lymphatic drainage. This nursing intervention facilitated the acquisition of knowledge regarding the prevention of lymphedema, instilled a strong motivation for prevention, and promoted the adoption of preventive health behaviors. Consequently, the risk of edema development and the occurrence of edema-related symptoms were reduced in the intervention group compared to the control group. Concurrently, patients received psychological counseling to enhance their psycho-emotional state, which significantly improved their physiological comfort. These favorable factors contributed to an enhancement in the quality of life of the patients.

There are several limitations to this study. First, the duration of the intervention was short because this study was limited by time factors that prevented continuation of follow-up, and later studies should extend the duration of the intervention to 1 year or even longer. Second, most patients received the intervention during the fall and winter months. It is not yet clear whether climate affects the occurrence of lymphedema; therefore, future studies should explore this factor in depth to further confirm the effectiveness of the intervention program. Third, the study population was derived from a single center only, and the total sample size of the study was relatively small and underrepresented.

## CRediT authorship contribution statement

D.W. X and W.F.S contributed to the conception of the study, Y.W contributed to the conception of the study and performed the data analyses and wrote the manuscript, L.T and S. W collected the data and helped to perform the data analysis. All authors had full access to all the data in the study, and the corresponding author had final responsibility for the decision to submit for publication. The corresponding author attests that all listed authors meet authorship criteria and that no others meeting the criteria have been omitted.

## Ethics statement

The study design was approved by the Ethics Committee of the Affiliated Hospital of Jiangnan University in Wuxi, Jiangsu, China (Approval No. JNMS042200183) and was conducted in accordance with the 1964 Helsinki Declaration and its later amendments or comparable ethical standards. All participants provided written informed consent.

## Funding

This work was supported by the Supports for Leading Talents in Medical and Health Profession (Mading academician, 4532001THMD), Beijing Bethune charitable Foundation (Grant No. 2022-YJ-085-J-Z-ZZ 011), Top Talent Support Program for Young and Middle-aged people of Wuxi Health Committee (Grant No. BJ2020047). The funders had no role in considering the study design or in the collection, analysis, interpretation of data, writing of the report, or decision to submit the article for publication.

## Data availability statement

The data that support the findings of this study are available from the corresponding author, Shi WF, upon reasonable request.

## Declaration of generative AI and AI-assisted technologies in the writing process

No AI tools/services were used during the preparation of this work.

## Declaration of competing interest

The authors declare no conflict of interest.

## References

[1] Dyba T, (2021). The European cancer burden in 2020: incidence and mortality estimates for 40 countries and 25 major cancers. Eur J Cancer.

[2] Rockson S.G. (2018). Lymphedema after breast cancer treatment. N Engl J Med.

[3] Al-Hilli Z., Wilkerson A. (2021). Breast surgery: management of postoperative complications following operations for breast cancer. Surg Clin North Am.

[4] Koelmeyer L.A., Gaitatzis K, Dietrich MS. (2022). Risk factors for breast cancer-related lymphedema in patients undergoing 3 years of prospective surveillance with intervention. Cancer.

[5] Zou L, (2018). The incidence and risk factors of related lymphedema for breast cancer survivors’ post-operation: a 2-year follow-up prospective cohort study. Breast Cancer.

[6] Abouelazayem M., Elkorety M., Monib S. (2021). Breast lymphedema after conservative breast surgery: an up-to-date systematic review. Clin Breast Cancer.

[7] Ribeiro Pereira A.C.P., Koifman R.J., Bergmann A. (2017). Incidence and risk factors of lymphedema after breast cancer treatment: 10 years of follow-up. Breast.

[8] Rafn B.S., Christensen J., Larsen A., Bloomquist K. (2022). Prospective surveillance for breast cancer-related Arm lymphedema: a systematic review and meta-analysis. J Clin Oncol.

[9] Hespe G.E., Nores G.G., Huang J.-J., Mehrara B.J. (2017). Pathophysiology of lymphedema-Is there a chance for medication treatment?. J Surg Oncol.

[10] Shi B, (2023). Effects of a lymphedema prevention program based on the theory of knowledge–attitude–practice on postoperative breast cancer patients: a randomized clinical trial. Cancer Med.

[11] Ridner S.H., Dietrich MS, Cowher MS (2019). A randomized trial evaluating bioimpedance spectroscopy versus tape measurement for the prevention of lymphedema following treatment for breast cancer: interim analysis. Ann Surg Oncol.

[12] Hayes S.C., Singh B., Reul-Hirche H. (2022). The effect of exercise for the prevention and treatment of cancer-related lymphedema: a systematic review with meta-analysis. Med Sci Sports Exerc.

[13] Zhang Z., Leong Bin Abdullah M.F.I., Shari N.I., Lu P. (2022). Acceptance and commitment therapy versus mindfulness-based stress reduction for newly diagnosed head and neck cancer patients: a randomized controlled trial assessing efficacy for positive psychology, depression, anxiety, and quality of life. PLoS One.

[14] Temur K., Kapucu S. (2019). The effectiveness of lymphedema self-management in the prevention of breast cancer-related lymphedema and quality of life: a randomized controlled trial. Eur J Oncol Nurs.

[15] Qin Y., Lu J., Li S. (2023). Knowledge attitude, and practice of breast cancer patients toward lymphedema complications: cross-sectional study. J Cancer Educ.

[16] Shirley D., Thibodeau L., Catz SL. (2018). Cessation-related information, motivation, and behavioral skills in smokers living with HIV. AIDS Care.

[17] Yao X., Zhang L., Du J., Gao L. (2021). Effect of information-motivation-behavioral model based on protection motivation theory on the psychological resilience and quality of life of patients with type 2 DM. Psychiatr Q.

[18] Guan J., Zhang Y., You S. (2023). Application of protection motivation theory in epidemic prevention in patients with respiratory diseases under the COVID-19 pandemic: a cross-sectional study. Clin Res J.

[19] Lin H., Chen M., Yun Q., Zhang L., Chang C. (2022). Protection motivation theory and smoking quitting intention: findings based on structural equation modelling and mediation analysis. BMC Public Health.

[20] Kowalski R.M., Black K.J. (2021). Protection motivation and the COVID-19 virus. Health Commun.

[21] Taştekin Ouyaba A., Çiçekoğlu Öztürk P. (2022). The effect of the information-motivation-behavioral skills (IMB) model variables on orthopedia nervosa behaviors of pregnant women. Eat Weight Disord.

[22] Ge J., Zhao S., Peng X. (2022). Analysis of the weight management behavior of Chinese pregnant women: an integration of the protection motivation theory and the information-motivation-behavioral skills model. Front Public Health.

[23] Pereira de Godoy J.M., Pereira de Godoy L.M., Godoy Guerreiro, M. de F (2020). Prevalence of subclinical systemic lymphedema in patients following treatment for breast cancer and association with body mass index. Cureus.

[24] Fu M.R., Axelrod D., Haber J. (2008). Breast-cancer-related lymphedema: information, symptoms, and risk-reduction behaviors. J Nurs Scholarsh.

[25] Fu M.R., Axelrod D., Guth AA. (2016). mHealth self-care interventions: managing symptoms following breast cancer treatment. mHealth.

[26] Fu M.R., Liu B., Qiu JM. (2024). The effects of daily-living risks on breast cancer-related lymphedema. Ann Surg Oncol.

[27] Liu F., Li F., Fu MR. (2021). Self-management strategies for risk reduction of subclinical and mild stage of breast cancer-related lymphedema: a longitudinal, quasi-experimental study. Cancer Nurs.

[28] Beaton D.E., Wright J.G., Katz J.N., Upper Extremity Collaborative Group (2005). Development of the QuickDASH: comparison of three item-reduction approaches. J Bone Joint Surg Am.

[29] Rafn B.S., McNeely M.L., Camp P.G., Midtgaard J., Campbell K.L. (2019). Self-measured Arm circumference in women with breast cancer is reliable and valid. Phys Ther.

[30] Alcorso J., Sherman K.A., Koelmeyer L., Mackie H., Boyages J. (2016). Psychosocial factors associated with adherence for self-management behaviors in women with breast cancer-related lymphedema. Support Care Cancer.

[31] Camerini A.-L., Diviani N., Fadda M., Schulz P.J. (2019). Using protection motivation theory to predict intention to adhere to official MMR vaccination recommendations in Switzerland. SSM Popul Health.

[32] Bruce J., Mazuquin B., Canaway A. (2021). Exercise versus usual care after non-reconstructive breast cancer surgery (UK PROSPER): multicentre randomised controlled trial and economic evaluation. Br Med J.

[33] Gao Bili, Yonghui Su, Lai Chunxiu (2023). The impact of health education on lymphedema prevention behavior and quality of life in breast cancer surgery patients. Chin J Health Stand Manag.

[34] Siqueira T.C., Frágoas S.P., Pelegrini A., de Oliveira A.R., da Luz C.M. (2021). Factors associated with upper limb dysfunction in breast cancer survivors. Supp Care Cancer.

[35] Xiao Yu, Yanmei Ma, Xu Mao, Sitong Guo, Lin Gao (2023). Evaluation of the effect of family-centered empowerment education on upper limb function rehabilitation in postoperative breast cancer patients. Gen Pract Nurs.

[36] Horváth A., Rédling M. (2022). Breast cancer-related lymphedema and treatment. Orv Hetil.

[37] Cal A., Bahar Z., Gorken I. (2020). Effects of Health Belief Model based nursing interventions offered at home visits on lymphedema prevention in women with breast cancer: a randomised controlled trial. J Clin Nurs.

[38] Cansız G., Arıkan Dönmez A., Kapucu S., Borman P. (2022). The effect of a self-management lymphedema education program on lymphedema, lymphedema-related symptoms, patient compliance, daily living activities and patient activation in patients with breast cancer-related lymphedema: a quasi-experimental study. Eur J Oncol Nurs.

[39] Thompson B., Gaitatzis K., Janse de Jonge X., Blackwell R., Koelmeyer L.A. (2021). Manual lymphatic drainage treatment for lymphedema: a systematic review of the literature. J Cancer Surviv.

[40] Qiao J., Yang LN., Kong YH. (2023). Effect of manual lymphatic drainage on breast cancer-related postmastectomy lymphedema: a meta-analysis of randomized controlled trials. Cancer Nurs.

[41] Szczuka Z., Siwa M., Abraham C. (2023). Handwashing adherence during the COVID-19 pandemic: a longitudinal study based on protection motivation theory. Soc Sci Med.

[42] Ranahan M., Dolph B., VonVisger J. (2021). A narrative review of qualitative studies describing access to kidney transplantation. Prog Transplant.

