# Supplementary material for: Effectiveness of a lymphedema prevention program for patients with breast cancer: A randomized controlled trial based on the Protection Motivation Theory and Information-Motivation-Behavioral Skills Model
Source: Asia Pac J Oncol Nurs. 2025 Feb 9;12:100667. doi: 10.1016/j.apjon.2025.100667 (PMC11926721; doi:10.1016/j.apjon.2025.100667)
Supplement: Multimedia component 1 [file mmc1.docx]

**PMT-IMB model-based intervention program implementation drafts**

| **intervention time** | **Information Support** | **Motivational support** | **Behavioral Skills Support** | **Forms of intervention** |
| --- | --- | --- | --- | --- |
| **First time in the group** | ① Through the Manual of Preventing Upper Limb Lymphedema on the Surgical Side, explain to patients the knowledge related to breast cancer and lymphedema, such as the causes of BCRL, risk factors, early symptoms and prevention methods, etc., to promote the patients' understanding of their own disease and the prevention of lymphedema. ② Provide personalized health education according to patients' own needs, and invite patients' family members to observe the learning. | ① Communicate with patients, establish a good relationship of trust, and guide patients to realize that axillary lymph node dissection sequential radiotherapy for breast cancer is a high-risk factor for the occurrence of lymphedema. ② Inform patients of the knowledge about lymphedema disease and the serious consequences of lymphedema by introducing adverse cases of prevention of lymphedema in breast cancer patients. (See below for specific communication). | ①Teach patients and their families to learn the correct measurement of the circumference of the upper limbs and lymphatic drainage techniques, inform patients and their families of the principles and sequence of lymphatic drainage, and at the same time, play video tutorials and conduct on-site teaching, so that the patients can learn to self-lymphatic drainage. ②Teach relaxation therapy, such as music therapy, so that patients can master self-relaxation techniques; encourage patients to participate in daily activities, such as singing or chatting with friends and relatives. | **face to face** |
| **First to second week** | ① Review what you learned last time, collect feedback from patients and give them personalized counseling. ② Provide new knowledge on prevention of lymphedema to strengthen patients' knowledge. ③Encourage patients to actively ask questions and discuss to deepen their understanding of the dangers of lymphedema and self-management. Answer any questions patients have. | ① Communicate with patients for the second time, inform patients of the prevalence and susceptibility of lymphedema, mobilize the enthusiasm of patients to prevent lymphedema, encourage patients to express their ideas about their own symptoms and the prevention of lymphedema, and assist patients to jointly develop preventive behavioral goals and behavioral plans. ②Family support, encourage family members to actively communicate with patients, support patients' preventive behavioral lifestyle, and enhance family members' psychological support for patients so that they can face life with a positive mindset. | ①Let the patients master the skills of self-lymphatic drainage, emphasizing that the drainage technique should be gentle and the speed should not be too fast, and inviting family members to participate in the whole process, so as to facilitate the supervision and cooperation of the patients. At the same time, tell the patients how to correctly carry out functional limb exercise, avoid exercise mistakes, and reduce the risk of lymphedema. ② Advise patients how to protect themselves in daily life, for example: use plant-based moisturizers, avoid mineral oil moisturizers; avoid sauna or hot bath; avoid scratches and infections on the upper limb skin on the operated side, and so on. | **WeChat platform or telephone** |
| **Third to fourth week** | ①Review what you learned last time, collect patient feedback, and provide personalized teleconsultation. ② Push the knowledge on the topic of prevention of lymphedema through the online platform and remind patients to study. At the same time, the Q&A content is pushed to the patient's questions so that the patient can learn. | ① Conduct a third communication with the patient to assess the patient's motivation at this stage and increase the patient's awareness of the benefits of lymphedema prevention behaviors; motivate the patient's self-confidence in adhering to healthy behaviors; explore the patient's coping methods for preventing lymphedema and encourage their active participation in preventing lymphedema, and ② Peer support, by sharing the preventive behavioral plans and experiences of the patients who have been effective in preventing lymphedema, so that they are able to encourage the patients' confidence to continue to maintain healthy behaviors. | ① Evaluate the patients' lymphatic drainage, for patients with poor compliance, emphasize the importance of self-lymphatic drainage to improve the prognosis and quality of life, tell the patients that they should insist on self-lymphatic drainage, and instruct the patients' family members to supervise the patients' preventive behaviors and record the implementation. ②Teach patients to master the self-checking method for early symptoms of lymphedema, such as numbness, heaviness or tightness in the upper limbs, swelling and redness of the skin of the breast or chest wall, and elevated skin temperature, etc., and inform them of the importance of checking the skin condition of the upper limbs on the operated side on a regular basis. | **WeChat platform or telephone** |
| **Fifth to eighth week** | ① Review the content of the last study and give targeted information support to patients with weak knowledge; collect patients' feedback and provide personalized remote consultation. ② Understand the implementation of patients' preventive behaviors, patiently answer patients' questions, and push new knowledge of lymphedema prevention to improve patients' awareness. | ① Communicate with the patient for the fourth time to assess the implementation of preventive behaviors during the patient's stay at home, help the patient to solve the difficulties encountered in the implementation of the plan, and improve the patient's self-confidence in being able to effectively adopt preventive behaviors on his/her own. Encourage the patient to continue to adhere to the prevention program. ② Reverse motivation of the patient to motivate preventive behaviors by informing the patient of the dangers of breast cancer lymphedema on life. | ①Enhance the patients' self-management ability, tell the patients to adhere to self-lymphatic drainage and record the implementation. By using pictures and video demonstrations and other forms of prevention of lymphedema behavioral education again. ② Actively give patients psychological guidance, instruct patients to learn self-psychological adjustment, such as breathing relaxation therapy: teach patients to breathe deeply and evenly, slowly bulge the abdomen when inhaling, and slowly retract the abdomen when exhaling. | **WeChat platform or telephone** |
| **Ninth to twelfth week** | ①Review the content of the last study, collect feedback from patients and answer questions. ② Push the related preventive knowledge explained by experts' video and consolidate the review. | ① Fifth motivational support: increase the patient's confidence in his or her ability to effectively adopt health behaviors. ② Assessing the difficulties and obstacles encountered by patients in overcoming the difficulties of adopting health behaviors, and discussing them with patients to help them reduce the difficulties encountered in making changes in health behaviors. | ① Inform patients of the importance of adhering to self-lymphatic drainage and strengthen their compliance. ② Psychological adjustment, encourage and patiently listen to the patient's expression, and give the patient psychological support. | **WeChat platform or telephone** |

**(1) Information support**

The first intervention mainly carried out personalized health education for patients face-to-face through the “Prevention of Postoperative Upper Extremity Lymphedema Brochure”, which was implemented mainly in wards or conference rooms. After the patients were discharged from the hospital at the end of the treatment, we mainly provided patients with knowledge related to the prevention of lymphedema through the WeChat platform, and we pushed the knowledge and reminded the patients to learn 2 times/week. If patients have any questions, they can leave a message in the background message box of the public number or contact us by phone. The researcher will summarize the questions raised by the patients, and the lymphedema nursing experts will answer the questions, and the researcher will make a unified response.

**(2) Motivational support**

**First motivational support**

**①Time:** The first day of enrollment

**②Theme:** Perceived threat, first building motivation

**③ Purpose:** Firstly, establish a good interpersonal relationship with the patient, inform the patient of the purpose and significance of this study; encourage the patient to express his/her views on the disease; assess the patient's knowledge of lymphedema; ask the patient how he/she can prevent lymphedema in normal times; and guide the patient to elaborate on the hazards that lymphedema can bring. Inform the patient of the importance of adhering to self-lymphatic drainage and completing records on time.

**④Communication content:** How do you think about breast cancer as a disease? Can you describe the current condition of the upper extremity on the operated side? Do you know anything about the complication of lymphedema? Do you know how to prevent lymphedema in daily life? Are you aware that lymphedema may occur after breast cancer treatment? Do you know what lymphatic drainage is? Do you know what are the benefits of adhering to lymphatic drainage? Questions were asked to enhance the patient's knowledge of lymphedema, the serious consequences of lymphedema on life, and the importance of lymphatic drainage. After the interview, stay in touch with the patient to provide follow-up support and follow up. Ensure that they are supported in coping with lymphedema and address issues and barriers that may arise.

**Second, motivational support**

**①Time:** 2nd week of enrollment

**②Theme:** Perceiving susceptibility, enhancing motivation

**③ Purpose:** To help patients recognize their susceptibility and enhance their motivation to adopt healthy behaviors, thus prompting them to take positive actions. Emphasize the importance of adhering to self-lymphatic drainage and completing the punch card as scheduled.

**④ Communication:** Are you aware of the risk factors for breast cancer lymphedema? Do you consider yourself susceptible? Has anyone mentioned the importance of preventing lymphedema during your breast cancer treatment? How do you feel about preventing lymphedema? Assess the patient's perception of susceptibility to lymphedema, encourage the patient to express their innermost thoughts, and provide advice and support according to the patient's needs. Share cases of other breast cancer patients who have successfully coped with lymphedema to inspire confidence and hope. Ask the patient about the implementation of self-lymphatic drainage, analyze the difficulties that need to be solved at present, the efforts that need to be made at present and how to ensure the implementation and completion of the plan.

**Third motivational support**

**①Time:** 4th week of enrollment

**②Theme:** Effective response, benefit a lot

**③ Purpose:** The main purpose is to help patients to be able to respond effectively to prevent lymphedema from occurring, to benefit from it, and to motivate patients' self-confidence in adhering to healthy behaviors; to explore patients' coping methods for preventing lymphedema and to encourage their active participation in the prevention of lymphedema in order to improve patients' quality of life. Reinforce the importance of preaching adherence to self-lymphatic drainage and completing the punch card as scheduled.

**④Communication:** Are you able to effectively take measures to prevent lymphedema? How much knowledge do you have about the prevention of lymphedema? How much do you know about self-directed lymphatic drainage? If you could see a positive effect of preventing lymphedema, how do you think it would affect your recovery and life? Discuss the positive consequences of an effective response to lymphedema, such as reduced swelling and discomfort, improved recovery outcomes and quality of life. Emphasize that positive outcomes increase patients' motivation and confidence; encourage them to consistently apply effective strategies in their daily lives and provide support and encouragement. Ask patients about self-implementation of lymphatic drainage and help them cope together if they encounter difficulties.

**Fourth motivational support**

**①Time:** 8th week of enrollment

**②Theme:** Motivate patients and enhance confidence

**③ Purpose:** To emphasize the importance of self-efficacy and enhance patients' confidence. To understand patients' confidence level in preventing lymphedema and provide encouragement and support to help patients develop positive beliefs and attitudes to promote effective health behaviors. Understand the implementation of the patient's prevention plan and explore with the patient their own personal abilities and resources to meet the challenges of lymphedema; learn about their past experiences in dealing with other difficulties and how these can be applied to lymphedema prevention efforts.

**④ Communication:** How confident are you in preventing lymphedema? Do you feel you have sufficient capacity and resources to prevent lymphedema? Have you recently completed your lymphedema prevention program on schedule? Have there been similar difficulties or challenges and how have you overcome them in the past? What do you consider to be your greatest strength in preventing lymphedema? How do you remind yourself to maintain positive beliefs and attitudes about the challenges of lymphedema in your daily life? Have you heard stories of other breast cancer patients who have successfully prevented lymphedema? How have these stories impacted your confidence? Ask questions such as assessing the patient's level of self-efficacy in preventing lymphedema, to understand the patient's perception of his or her ability to effectively prevent lymphedema, and the factors that may affect the patient's confidence; explain the concept of self-efficacy to the patient. Emphasizing that self-efficacy is critical to success in coping with lymphedema. Encourage them to believe in their own abilities. Finally, review the patients' progress in coping with lymphedema and highlight their efforts and achievements; emphasize the self-efficacy they have already demonstrated to motivate them to continue their efforts and believe in their ability to effectively prevent lymphedema.

**Fifth motivational support**

**①Time:** 12th week of enrollment

**②Theme:** Overcoming difficulties, victory is in sight

**③ Purpose:** to analyze and overcome the obstacles encountered in the process of preventing lymphedema with the patient, to understand the specific difficulties encountered by the patient, the completion of the implementation plan, and to provide support and advice to help the patient develop coping strategies to achieve the goal of preventing lymphedema; to summarize the problems encountered in the process of implementing health behaviors, and to help the patient to overcome the disadvantages of implementing health behaviors.

**④Communication content:** What is the biggest obstacle you are currently facing in preventing lymphedema? Do you feel a lack of knowledge or information to effectively prevent lymphedema? Do you encounter time constraints or other commitments in your daily life that make it difficult to make time for lymphedema prevention activities? What specific difficulties or challenges have you encountered in past attempts to prevent lymphedema? Questions such as Explore specific obstacles patients face, such as lack of information, time constraints, or psychological barriers, and understand how these obstacles affect and limit them; identify patients' priority obstacles, and work with patients to identify the most important obstacles that may have the greatest impact on their lymphedema prevention efforts. Work with patients to develop individualized coping strategies to help them overcome specific impediments based on their circumstances and abilities. Provide information and knowledge based on the specific obstacles patients face to help them understand how to overcome them. Provide ongoing support and follow-up to ensure that patients can always seek support and counseling for any challenges in the prevention of lymphedema.

**(3) Behavioral skills intervention**

After providing information support to the patients, behavioral skills support is given to the patients, mainly including the patients can master the self-lymphatic drainage technique and insist on its implementation, and record the implementation situation at the same time; they can master the correct functional exercise techniques and how to protect the affected limbs to avoid damages; they can teach the patients self-relaxation techniques, such as music therapy and respiratory relaxation therapy, so that the patients can better master the health behaviors by themselves.

**Nursing health education in the control group**

| **(1) Daily aspects** | a. Advise patients to maintain good habits in daily life, maintain a healthy diet, avoid excessive salt intake, maintain an appropriate body weight, avoid obesity, and engage in moderate exercise on a regular basis.  b. Avoid overly strenuous activities to reduce the burden on the lymphatic system. Also, avoid maintaining the same posture for a long time: maintaining the same posture for a long time may affect the flow of lymphatic fluid and increase the risk of lymphoedema. Therefore, when sitting or standing for long periods of time, you should adjust your posture or take appropriate breaks when appropriate.  c. Perform lymphatic drainage massage, seek guidance from nursing professionals, learn proper lymphatic drainage massage techniques, and perform regular massage to promote lymphatic fluid flow.  d. After radiotherapy for breast cancer, if swelling, tenderness or other symptoms related to lymphoedema occur, consult your doctor or professional caregiver for appropriate assessment and treatment advice. |
| --- | --- |
| **(2) Skin Care** | a. Prior to starting radiotherapy, assess and examine the patient's skin and instruct the patient to clean the irradiated area properly. Patients should avoid using hot and soapy water to clean the area to prevent skin damage or the radiotherapy markings from becoming blurred, which may affect the effectiveness of radiotherapy.  b. Advise patients to avoid the use of irritating disinfectants after radiotherapy and not to scratch the skin of the irradiated area.  c. Instruct the patient to remove all metal items worn, such as earrings, keys, watches, etc., to prevent these items from increasing the absorption of radiation.  d. If there is heat or flushing of the skin, it is recommended to wear loose and comfortable clothing to minimize friction that can cause irritation to the damaged skin. |
| **(3) Psychological care** | Breast cancer is a chronic disease, and the disease itself and long-term treatment make patients feel nervous and anxious. Nursing staff listen to patients' emotional expression, provide emotional support and comfort, make patients feel understood and supported, help patients adjust negative thinking, relieve anxiety and fear, so that patients can maintain a good psychological state and quality of life during the treatment process. |
| **(4) Follow-up care** | For patients who have been discharged from the hospital, regular follow-ups are carried out, inquiring about the current physical state of the patient, answering questions raised by the patient, informing the patient of the relevant precautions, providing relevant guidance for the patient, and informing the patient of the next radiotherapy time. |

**WeChat Official Account Push Content**

| Push topic | Push topic content | Content format |
| --- | --- | --- |
| Knowledge of Preventing Lymphedema | (1) Stages of Lymphedema.  (2) Lymphedema Q&A.  (3) Early Identification of Lymphedema.  (4) Key Points for Preventing Lymphedema.  (5) Common Misconceptions in Preventing Lymphedema. | (1) Image and text push  (2) Video display |
| motivational support | (1) The severity of lymphedema (2) Susceptibility to lymphedema. (3) The benefits of healthy behaviors. (4) Successful Patient Experience Sharing. (5) Provide psychological support. | (1) Image and text push  (2) Video display |
| Behavioral Skills Support | (1) Self-lymphatic drainage.  (2) Skin protection. (3) Correct functional segment practice. (4) Music therapy. (5) Relaxation therapy. | (1) Image and text push  (2) Video display |
