# Supplementary material for: Effectiveness of a lymphedema prevention program for patients with breast cancer: A randomized controlled trial based on the Protection Motivation Theory and Information-Motivation-Behavioral Skills Model
Source: Asia Pac J Oncol Nurs. 2025 Feb 9;12:100667. doi: 10.1016/j.apjon.2025.100667 (PMC11926721; doi:10.1016/j.apjon.2025.100667)
Supplement: Multimedia component 2 [file mmc2.docx]

**Experts Correspondence**

In this study, 10 representative experts were selected to participate in the revision of the protocol of this study through purposive sampling method. After the experts' consent, we sent the first draft of the intervention protocol to the experts for review. The inclusion criteria of the experts were: the research field was related to oncology nursing, lymphedema nursing and psychological nursing; the education level was above bachelor's degree; the title was above intermediate level; the working experience must be above 10 years, and they had an in-depth knowledge of the treatment and nursing in this research field. The specific experts included are as follows:

Table 1-1 Basic information on experts

| Specialist No. | title | Academic qualifications | Research direction | Years of experience |
| --- | --- | --- | --- | --- |
| 1 | chief physician | doctorate | Tumor Radiotherapy | 24 |
| 2 | chief physician | doctorate | Breast Surgery | 20 |
| 3 | deputy chief physician | doctorate | Breast Surgery | 15 |
| 4 | Chief Nurse | master's degree | Tumor Care | 17 |
| 5 | Chief Nurse | master's degree | Tumor Care | 18 |
| 6 | Chief Nurse | undergraduate | Nursing management | 27 |
| 7 | nurse practitioner-in-charge | undergraduate | Tumor Care | 11 |
| 8 | nurse practitioner-in-charge | undergraduate | Lymphedema Therapist | 15 |
| 9 | chief physician | master's degree | psychology | 19 |
| 10 | nurse practitioner-in-charge | undergraduate | psychological care | 10 |

**Degree of activeness in consulting experts**

A total of 10 experts were invited for consultation for this study and finally feedback and comments were received from 10 experts. The effective recall rate was 100%.

**Degree of authority of the consulting expert**

The calculation of the expert authority coefficient (Cr) depends on the expert's familiarity with the content (Cs) and the expert's basis for judging the program (Ca), which is calculated by the formula: (Cr=Ca+Cs)/2. Through the analysis of the recovered information, it is calculated that Cs=0.80, Ca=0.93, and Cr=0.87. An expert authority coefficient of ≥0.7 indicates that the expert's degree of authority is high.

Table 1-2 Self-assessment of experts' familiarity with program content (Cs)

| Cs | Familiar | More familiar | Fairly familiar | Less familiar | Unfamiliar Not familiar |
| --- | --- | --- | --- | --- | --- |
| Knowledge of Lymphedema Prevention | 0.9（7） | 0.7（2） | 0.5（1） | 0.3（0） | 0.1（0） |
| Lymphoedema care | 0.9（7） | 0.7（1） | 0.5（2） | 0.3（0） | 0.1（0） |
| PMT-IMB Theory and Application | 0.9（6） | 0.7（2） | 0.5（2） | 0.3（0） | 0.1（0） |

*Note:*

*Cr, the expert authority coefficient is a measure of an expert's level of knowledge and experience in a given field, which reflects the degree of authority of the expert on the issue being consulted.*

*Cr is determined by two factors: the expert's familiarity with the problem (Cs) and the expert's basis for judging the indicator (Ca). Cs, denotes the expert's familiarity with the entry; Ca, denotes the expert judgment coefficient.*

*A Cr of 0.5 or more is generally considered to indicate a high degree of expert authority, while a Cr greater than or equal to 0.7 is considered acceptable.*

Table 1-3 Self-assessment of the basis for expert judgment (Ca)

| Basis of judgment |  | Assignment of the basis of judgment |  |
| --- | --- | --- | --- |
|  | Large | Medium | Small |
| practical experience | 0.5（7） | 0.4（3） | 0.3（0） |
| theoretical analysis | 0.3（7） | 0.2（3） | 0.1（0） |
| Reference to national and international literature | 0.1（6） | 0.1（3） | 0.1（1） |
| subjective perception | 0.1（0） | 0.1（3） | 0.1（7） |

*Note:* *Ca, Self-assessment of the basis for expert judgment*

**The expert modification recommendations are as follows:**

(1) The nursing intervention program should fully integrate the PMT-IMB theory into the implemented nursing intervention program to make the intervention program more scientific, reasonable and applicable.

(2) The content of the intervention program should have a certain degree of professionalism and rigor, and the intervention team must be supervised and managed by the graduate supervisor.

(3) The content of the intervention should be more specific and detailed, which will help the understanding of the program; the topic of the interview should be clarified before conducting the motivational interview.

(4) Enhance patients' motivation to prevent lymphedema through a multidimensional approach based on the PMT-IMB model, thereby enabling patients to generate and adhere to preventive behaviors on their own.

(5) The online intervention content push should be clear and easy to understand, and patients should be strengthened to be reminded to study, so as to ensure the learning efficiency of patients.
