# Supplementary material for: Effectiveness of a lymphedema prevention program for patients with breast cancer: A randomized controlled trial based on the Protection Motivation Theory and Information-Motivation-Behavioral Skills Model
Source: Asia Pac J Oncol Nurs. 2025 Feb 9;12:100667. doi: 10.1016/j.apjon.2025.100667 (PMC11926721; doi:10.1016/j.apjon.2025.100667)
Supplement: Multimedia component 3 [file mmc3.docx]

| **adverse event** | **Processing measures** |
| --- | --- |
| Breast cancer patients with irregularities in measuring the circumference of the upper extremities using soft tape measurements, resulting in biased results. (Usually occurs in the first to second week) | The researchers had patients take measurements on site and demonstrated live, face-to-face, teaching patients and their families the standardized method of measuring the circumference of the upper limb. |
| Breast cancer self-drainage maneuvers are not standardized and are too strong or too light, leading to poor results. (Usually occurs in the third to fourth week of intervention) | The lymphoedema drainers performed manipulative drainage operations on patients on a one-to-one basis and checked their manipulation techniques and degree of standardization on the spot and gave timely explanations and teaching for incorrect operations. |
| Breast cancer patients lack confidence and fail to adhere to functional exercise drills and routine measurement procedures when at home. (Usually occurs between weeks 5 and 12) | The researchers urged patients to adhere to lymphoedema-related preventive behaviors through five sessions of motivational support and to complete upper to circumferential diameter measurements in order to improve patients' preventive behavior change. |
